# The effect of adult attachment on mobile phone dependence among university students: the mediating role of loneliness

**DOI:** 10.3389/fpsyg.2024.1494262

**Published:** 2024-12-18

**Authors:** Zhenhong Wang, Bin Xuan

**Affiliations:** ^1^School of Educational Science, Anhui Normal University, Wuhu, China; ^2^College of Education, Fuyang Normal University, Fuyang, China

**Keywords:** mobile phone dependence, adult attachment, loneliness, attachment anxiety, attachment avoidance

## Abstract

**Objective:**

This study aimed to examine the direct relationship between adult attachment and mobile phone dependence, as well as the mediating role of loneliness.

**Methods:**

Using a cross-sectional study design, 596 Chinese university students (mean age = 19.8, SD = 1.09; females = 309) completed the Experience in Close Relationship Inventory, the UCLA Loneliness Scale, and the Mobile Phone Addiction Index.

**Results:**

There were significant differences in loneliness and mobile phone dependence among university students with different adult attachment types. Adult attachment was found to have a significant positive correlation with mobile phone dependence. Moreover, adult attachment had an indirect association with mobile phone dependence through the mediating role of loneliness.

**Conclusion:**

Adult attachment and mobile phone dependence are closely related, and loneliness plays a vital role in this relationship, suggesting that mobile phone dependence can be reduced by alleviating university students’ loneliness. These findings enhanced the understanding of the mechanism of mobile phone dependence and provided new perspectives for the prevention and treatment of mobile phone dependence among university students.

## 1 Introduction

Mobile phone dependence refers to excessive engagement in activities related to mobile phones, characterized by solid cravings and persistent reliance on the device (Yen et al., 2009). This dependence can lead to a loss of self-control and negatively affect psychological and social well-being. Mobile phone dependence can contribute to various social and psychological issues among university students, including social isolation, decreased interpersonal skills, academic burnout, social anxiety, sleep disturbances, increased depression, frustration, and decreased commitment to studying (Al-Kandari and Al-Sejari, 2021; You et al., 2019; Luo et al., 2022; Shi et al., 2022; Yu et al., 2022; Chen et al., 2022; Zhu et al., 2022). In China, university students represent a significant demographic of mobile phone Internet users, as mobile devices have become essential tools for social interaction, daily life, and academic activities. However, various risk factors contribute to a concerning prevalence of mobile phone dependence within this group (Wang, 2022). Consequently, many studies have focused on understanding the causes and mechanisms behind mobile phone dependence in this demographic. Adult attachment and loneliness were found to be significant influences on university students’ mobile phone dependence (Luo and Hu, 2022). Previous research has indicated that adult attachment significantly impacts loneliness and can increase mobile phone dependence among university students (Liang et al., 2021; Li et al., 2021). Nevertheless, the role of loneliness in the relationship between adult attachment and mobile phone dependence has yet to be extensively explored.

Adult attachment is the emotional bond that promotes intimacy and security between an individual and their romantic partner, lover, or close friend. This bond is rooted in the emotional interactions experienced during childhood with a primary caregiver (Sable, 2008). Two orthogonal dimensions tap individual differences in adult attachment (Griffin and Bartholomew, 1994). Attachment anxiety is characterized by a heightened sense of insecurity and worry experienced by an individual within an intimate relationship (Hazan and Shaver, 1994). This anxiety often stems from uncertainties regarding a partner’s availability, responsiveness, and commitment. Conversely, attachment avoidance manifests as a defensive attitude and behavior that individuals display in the context of intimate relationships. This avoidance may arise from maladaptive attachment patterns developed during early experiences, causing the individual to feel uncomfortable with forming and maintaining close connections. Research has shown a significant correlation between adult attachment and mobile phone dependence among university students (Yao and Zhao, 2021). Students with a high level of attachment anxiety tend to use their mobile phones frequently to stay connected with their attachment partners. This behavior stems from a desire for security in the face of unstable relationships, feelings of insecurity, and a fear of abandonment, which can increase the risk of becoming dependent on their mobile phones. Conversely, those with high attachment avoidance often reject close connections as a way to protect themselves from their attachment needs. Due to a lack of interpersonal trust, they may struggle to form close relationships in real life, leading them to turn to virtual networks to alleviate their inner pain, which can further heighten the likelihood of mobile phone dependence (Parent, 2019). Accordingly, hypothesis one is proposed: adult attachment may significantly and positively predicted mobile phone dependence.

Attachment anxiety and attachment avoidance are significant predictors of loneliness (Liu et al., 2021; Wei et al., 2005; Ong et al., 2016). According to attachment theory, the early attachment relationship between a mother and infant shapes how individuals interact with others in adulthood (Fearon and Roisman, 2017). During their early development, university students are more likely to develop insecure attachments if their attachment figures are inaccessible or insensitive. Insecurely attached university students tend to experience higher levels of loneliness than those with secure attachments (Flaherty and Sadler, 2011). Students with high levels of attachment anxiety often struggle with a negative self-image and fear abandonment in their intimate relationships. To avoid feeling ignored, they may frequently send messages, make phone calls, or ask those around them how they are feeling. However, these behaviors can sometimes overwhelm others and lead to feelings of alienation in relationships, ultimately worsening their sense of loneliness (Nielsen et al., 2017). On the other hand, university students exhibiting high levels of attachment avoidance tend to shun attachment to others, leading to social withdrawal. They may be more inclined to socialize via the Internet than face-to-face interactions. Online socialization provides a certain level of anonymity and distance that allows them to stay in touch with others while remaining evasive. However, this virtual way of socializing may not fully satisfy their emotional needs, but rather exacerbate feelings of loneliness (Tejada et al., 2017).

Loneliness is a negative experience that arises from the lack of satisfactory relationships (Feng and Zhang, 2015). Research has demonstrated that loneliness significantly predicts mobile phone dependence (Wu et al., 2017; Li et al., 2023). According to social needs theory, loneliness arises as a response to unmet social needs (DiTommaso and Spinner, 1997). University students who experience feelings of intense loneliness are more likely to turn to the Internet. This can lead to dependence on mobile phones due to excessive internet use, which in turn negatively impacts their academic self-efficacy and academic achievement (Ramli et al., 2024; Mizani et al., 2022). As social networks expand and online communication patterns evolve, these students with heightened feelings of loneliness are increasingly drawn to online interactive social activities. They seek a sense of belonging, companionship, and security (Morahan-Martin, 1999). Additionally, the cognitive-behavioral model of problematic internet use suggests that lonely university students may have distorted perceptions of themselves and the world around them. They often feel dissatisfied with real-world relationships, develop an aversion to reality, and may use the Internet to escape loneliness. Unfortunately, this behavior can increase the risk of mobile phone dependence (Davis, 2001). Thus, hypothesis two is proposed: loneliness may play a mediating role in the relationship between adult attachment and mobile phone dependence.

Previous studies have investigated the connection between adult attachment and mobile phone dependence. However, the specific mediating role of loneliness in this relationship has yet to be thoroughly examined. Mobile phone dependence can result in both physical and mental harm for university students. Therefore, exploring the factors and mechanisms contributing to mobile phone dependence among university students is crucial to establish a theoretical foundation for effective interventions. The study has significant theoretical value and practical implications. This research offers a new perspective on the complex relationship between adult attachment, loneliness, and mobile phone dependence, enhancing the theoretical framework in related fields. Importantly, introducing loneliness as a mediating variable deepens our understanding of how these psychological phenomena interact. From a practical standpoint, universities can implement targeted mental health education initiatives that address loneliness among students. This could reduce excessive mobile phone dependence and improve overall mental health and quality of life. Additionally, student management departments could develop more personalized strategies tailored to students’ attachment styles and levels of loneliness. This approach would assist students in forming healthy social networks and maintaining balanced mobile phone usage habits.

## 2 Method

### 2.1 Participants and procedure

The study employed a convenience sample of 596 university students from four universities in China (mean age = 19.8, SD = 1.09; females = 309). Of the participants, 248 (41.61%) were only children, while 348 (58.39%) were non-only children. Regarding the grade, 172 (28.86%) were freshmen, 135 (22.65%) were sophomores, 150 (25.17%) were juniors, and 139 (23.32%) were seniors (Table 1). The study received approval from the Ethics Committee of the Faculty of Education at Fuyang Normal University (Ethics number: jyxy-2023-06-14-1). All participants provided consent to participate in the survey, which was conducted online. They were assured that the data would be used exclusively for research purposes.

**Table 1:** Descriptive table of participant characteristics.

Variables	Categories	Number of samples	Percentage (%)
Gender	Male	287	48.15
	Female	309	51.85
Only Child	Yes	248	41.61
	No	348	58.39
Grade	Freshman	172	28.86
	Sophomore	135	22.65
	Junior	150	25.17
	Senior	139	23.32

### 2.2 Measures

#### 2.2.1 Experience in close relationship inventory

The Experience in close relationships inventory was developed in Brennan (1998), and the Chinese version was revised in Li and Kato (2006). This scale comprises 36 questions categorized into two dimensions: attachment anxiety and attachment avoidance. The items are scored on a 7-point Likert scale, ranging from “strongly disagree” to “strongly agree.” In the context of this study, Cronbach’s alpha coefficient for scores from the attachment avoidance dimension was 0.90, while Cronbach’s alpha coefficient for scores from the attachment anxiety dimension was 0.87.

#### 2.2.2 UCLA loneliness scale

The UCLA loneliness scale was developed in Russell et al. (1978), and the Chinese version was revised in Liu (1999). The items are scored on a 4-point Likert scale, ranging from “never” to “always.” A higher score on the scale signifies a more robust experience of loneliness. In the context of this study, the Cronbach’s alpha coefficient for scores from this scale was 0.91.

#### 2.2.3 Mobile phone addiction index

The mobile phone addiction index was developed in Leung (2008), and the Chinese version was revised in Huang et al. (2014). It comprises 17 questions encompassing four dimensions: loss of control, withdrawal, avoidance, and ineffectiveness. The items are scored on a 5-point Likert scale, ranging from “rarely” to “always.” In the context of this study, the Cronbach’s alpha coefficient for the scores from this scale was 0.89.

### 2.3 Data analysis

We conducted descriptive analysis, internal consistency checks, differential analysis, Pearson’s correlation analysis, and a common method bias test using SPSS 26.0. To test the model, we utilized Andrew Hayes’s Process macro for SPSS (Hayes, 2013). For evaluating mediation, we performed a bias-corrected bootstrap estimation with 5,000 samples and established a 95% confidence interval (CI). Mediation is considered significant if the confidence interval does not include zero.

## 3 Results

### 3.1 Common method biases test

Harman’s single-factor test addressed the risk of common method bias in this study (Zhou and Long, 2004). The results showed that the variance explained by the first factor was 21.60%, which is below the acceptable threshold of 40%. Therefore, this indicates no significant methodological bias in the study.

### 3.2 Analysis of variances

Based on the classification criteria for adult attachment types (Shaver and Fraley, 2004), 596 university students were categorized into four distinct attachment types. Among these participants, 399 students (66.9%) were identified as having a secure attachment style. Additionally, 113 students (19.0%) exhibited a preoccupied attachment, 54 students (9.1%) demonstrated a dismissing-avoidant attachment, and 30 students (5.0%) showed a fearful-avoidant attachment style.

A one-way analysis of variance was conducted to examine the differences in loneliness and mobile phone dependence among university students with varying attachment styles. As shown in Table 2, significant differences in loneliness were observed among students categorized by their attachment styles (*F* = 45.193, *p* < 0.001). *Post hoc* analysis revealed that students with secure attachment exhibited significantly lower levels of loneliness compared to those with dismissing-avoidant, preoccupied, and fearful-avoidant attachment styles. Furthermore, there were significant differences in mobile phone dependence among university students with different attachment styles (*F* = 22.209, *p* < 0.001). Post hoc testing indicated that students with secure attachment had significantly lower mobile phone dependence than those with preoccupied and fearful-avoidant attachment styles. Additionally, students with preoccupied and fearful-avoidant attachment styles demonstrated significantly higher mobile phone dependence than those with dismissing-avoidant attachment.

**Table 2:** Analysis of variances (*N* = 596).

Variables	Loneliness			Mobile phone dependence		
	*M*	*SD*	*F*	*M*	*SD*	*F*
Secure	1.91	0.47	45.193^***^	2.32	0.63	22.209^***^
Preoccupied	2.37	0.37		2.85	0.62	
Dismissing-avoidant	2.22	0.47		2.44	0.61	
Fearful-avoidant	2.54	0.32		2.73	0.77	

^***^p < 0.001.

### 3.3 The correlation between the study variables

Table 3 shows the means, standard deviations, and correlation coefficients of adult attachment, loneliness, and mobile phone dependence. The results showed that all the variables had significant correlations with each other. Specifically, attachment anxiety significantly and positively correlated with loneliness (*r* = 0.523, *p* < 0.01), and attachment avoidance significantly and positively correlated with loneliness (*r* = 0.397, *p* < 0.01). Furthermore, attachment anxiety significantly and positively correlated with mobile phone dependence (*r* = 0.491, *p* < 0.01), and attachment avoidance significantly and positively correlated with mobile phone dependence (*r* = 0.122, *p* < 0.01). Lastly, loneliness significantly and positively correlated with mobile phone dependence (*r* = 0.379, *p* < 0.01).

**Table 3:** Descriptive statistics and interrelations among all variables (*N* = 596).

Variables	*M*	*SD*	Attachment anxiety	Attachment avoidance	Loneliness	Mobile phone dependence
Attachment anxiety	3.34	0.97	1			
Attachment avoidance	3.20	0.86	0.338^**^	1		
Loneliness	2.06	0.50	0.523^**^	0.397^**^	1	
Mobile phone dependence	2.46	0.67	0.491^**^	0.122^**^	0.379^**^	1

^**^p < 0.01.

### 3.4 Mediation effect test

To explore the mediating role of loneliness in the relationship between adult attachment and mobile phone dependence among university students, the bias-corrected percentile Bootstrap method (with a resampling number of 5,000) was used to mediate the effects of the data using Model 4 in the macro program PROCESS 3.0, with a 95% confidence level for the confidence interval.

**Figure 1:**
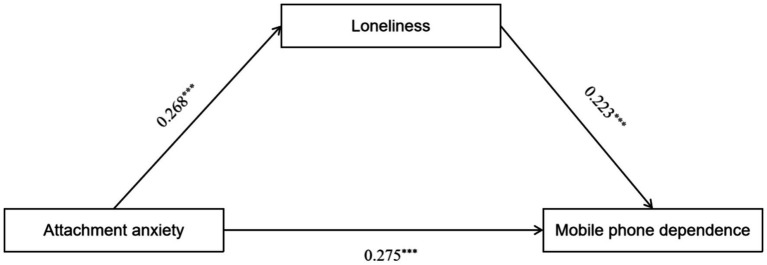
A test of the mediating role of loneliness between attachment anxiety and mobile phone dependence.

**Figure 2:**
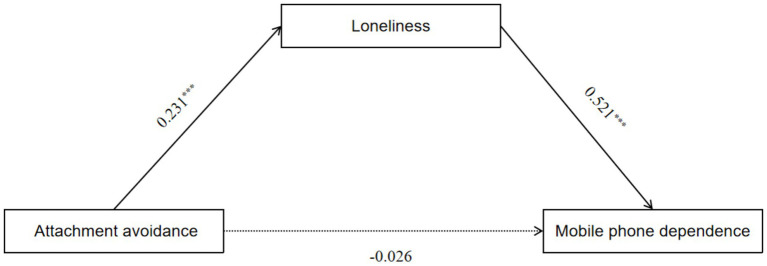
A test of the mediating role of loneliness between attachment avoidance and mobile phone dependence.

As illustrated in Figure 1, the mediation regression analysis indicated that attachment anxiety significantly and positively predicted mobile phone dependence (*β* = 0.275, *p* < 0.001). Furthermore, attachment anxiety significantly and positively predicted loneliness (*β* = 0.268, *p* < 0.001), and loneliness also significantly and positively predicted mobile phone dependence (*β* = 0.223, *p* < 0.001). In Figure 2, the mediation regression analysis demonstrated that attachment avoidance significantly and positively predicted loneliness (*β* = 0.231, *p* < 0.001). Additionally, loneliness significantly and positively predicted mobile phone dependence (*β* = 0.521, *p* < 0.001); however, attachment avoidance did not significantly predict mobile phone dependence (*β* = −0.026, *p* > 0.05).

Bootstrapping procedures with 5,000 resamples were conducted using a 95% bias-corrected confidence interval (CI) to further assess the significance of the mediation effects. The mediation effects were considered significant if the 95% CI for the path coefficient did not include zero. The results of the mediation analysis, presented in Table 4, indicate that loneliness partially mediates the relationship between attachment anxiety and mobile phone dependence. This conclusion is supported by the significance of the total effect, the mediating effect, and the direct effect identified in the analysis. In contrast, when examining the mediation of loneliness in the relationship between attachment avoidance and mobile phone dependence among university students, we found that both the total effect and the mediating effect were significant. However, the direct effect was not significant. This suggests that loneliness fully mediates the relationship between attachment avoidance and mobile phone dependence.

**Table 4:** Bootstrap analysis of mediation effects.

Type of effect	Model pathways	Effect size	Boot SE	Boot CI lower	Boot CI upper
Total effect	AAN → MPD	0.334	0.024	0.286	0.382
	AAV → MPD	0.094	0.032	0.032	0.156
Direct effect	AAN → MPD	0.275	0.028	0.219	0.329
	AAV → MPD	−0.026	0.032	−0.088	0.037
Indirect effect	AAN → L → MPD	0.059	0.018	0.026	0.095
	AAV → L → MPD	0.120	0.018	0.085	0.157

SE, standard error; CI, confidence interval; AAN, attachment anxiety; AAV, attachment avoidance; L, Loneliness; MPD, mobile phone dependence.

## 4 Discussion

### 4.1 Differential analysis of loneliness and mobile phone dependence

The loneliness experienced by university students with insecure attachment styles is significantly greater than that of their securely attached peers, as supported by previous studies (Zhang and Xu, 2014; DiTommaso et al., 2003). Students with insecure attachments often face a higher degree of attachment-related trauma, including experiences of abuse and neglect. As a result, they frequently struggle with trust and security in their interpersonal relationships, making it difficult for them to form stable and supportive connections. This, in turn, leads to increased feelings of loneliness.

Except for those with dismissing-avoidant attachment, university students with insecure attachment styles demonstrated significantly higher dependence on mobile phones compared to securely attached students. This finding aligns with prior studies (Gui et al., 2021; Konok et al., 2016). According to attachment theory, addictive or dependent behaviors may serve as a means of fulfilling an individual’s unmet attachment needs (Liese et al., 2020). In the face of painful or stressful situations, individuals with insecure attachment often resort to substances or specific behaviors to satisfy these needs, which remain unfulfilled by those in their close relationships. In contrast, university students with dismissing-avoidant attachment do not show a higher level of mobile phone dependence than their securely attached peers. This may be due to their ability to cope with unsatisfactory interpersonal relationships, which is a defense mechanism against their attachment needs (Kim and Koh, 2018).

### 4.2 The mediating role of loneliness

This study found that attachment anxiety and avoidance significantly and positively predicted mobile phone dependence, supporting Hypothesis 1. According to the uses and gratifications theory, when individuals’ needs are satisfied using a specific object or medium, this satisfaction motivates them to continue using it (Blumler and Katz, 1974). University students who experience high levels of attachment anxiety and avoidance often struggle to fulfill their attachment needs in genuine intimate relationships. As a result, they may turn to addictive or dependent behaviors as a way to compensate for these unmet needs. Mobile phones, which serve as tools for maintaining relationships, are particularly susceptible to being used in this compensatory manner, increasing the likelihood of mobile phone dependence (Veissière and Stendel, 2018).

This study indicated that loneliness partially mediated the relationship between attachment anxiety and mobile phone dependence among university students, supporting Hypothesis 2. Students with high levels of attachment anxiety often experience unstable responses from their attachment figures during their early development. As a result, they frequently worry about the availability of these figures and tend to adopt strategies that over-activate their attachment needs (Liu et al., 2021). In their current relationships with attachment figures, they grapple with feelings of insecurity, fear of abandonment, and profound loneliness. Consequently, they have a heightened desire for attachment and attempt continuously to remain connected to their attachment figures (Han et al., 2017). This urge leads university students with high attachment anxiety to constantly communicate with these figures via mobile phones, thereby increasing their risk of developing mobile phone dependence (Zhang et al., 2022).

Furthermore, loneliness mediated the relationship between attachment avoidance and mobile phone dependence among university students, supporting Hypothesis 2. Those with high attachment avoidance often experience increased feelings of loneliness, primarily due to their distrust of others and fear of rejection (Mikulincer and Shaver, 2003). The loner hypothesis suggests that individuals who see themselves as loners are particularly attracted to specific aspects of synchronous online social interactions, lacking conventional face-to-face interactions (Morahan-Martin and Schumacher, 2000; Jones and Moore, 1987). These online interactions offer enhanced self-expression, greater anonymity, and reduced social risks, making them especially appealing to highly attachment-avoidant university students. As a result, these individuals may increasingly rely on online social interactions or other forms of human-computer interaction to alleviate their loneliness, often arising from heightened social anxiety in real-life situations (Caplan, 2003). This tendency can lead to more frequent and prolonged mobile phone use, ultimately resulting in mobile phone dependence.

## 5 Limitations and future study

The study has certain limitations. First, using a convenience sample limits the generalizability of the findings. Second, relying on survey data may introduce unknown social desirability bias. Additionally, there is a need for longitudinal tracking data to assess causal relationships and dynamic shifts among variables accurately. Lastly, Harman’s single-factor test is inadequate in detecting common methods of bias evaluation.

Future research should utilize a probabilistic sampling method and a longitudinal design to monitor changes among university students over time, which would yield more definitive results. Moreover, more sophisticated research designs and alternative techniques, such as marker variable techniques, are recommended for identifying and addressing common methods of bias evaluation (Howard et al., 2024).

## Data Availability

The original contributions presented in the study are included in the article/supplementary material, further inquiries can be directed to the corresponding author.
